# Immediate salbutamol responsiveness does not predict long-term benefits of indacaterol in patients with chronic obstructive pulmonary disease

**DOI:** 10.1186/s12890-017-0372-z

**Published:** 2017-01-31

**Authors:** Pierre-Régis Burgel, Vincent Le Gros, Laurent Decuypère, Isabelle Bourdeix, Thierry Perez, Gaëtan Deslée

**Affiliations:** 1 Respiratory Medicine, APHP–Hôpital Cochin–Université Paris Descartes, 27 rue du Faubourg St Jacques, Sorbonne Paris Cité, Paris, 75014 France; 2 Respiratory Medical Department, Novartis Pharma SAS, Rueil-Malmaison, Paris, France; 3 Pulmonary Department, CHU de Lille, Université de Lille, Lille, France; 4 Respiratory Medicine, INSERM UMRS 903, Hôpital Maison Blanche–CHU de Reims, Reims, France

**Keywords:** COPD, Indacaterol, Reversibility, Salbutamol

## Abstract

**Background:**

The purpose of this study was to evaluate the correlation between immediate responsiveness with the short-acting β_2_-agonist salbutamol and effects of treatment with the ultra-long-acting β_2_-agonist indacaterol in patients with chronic obstructive pulmonary disease (COPD).

**Methods:**

The REVERBREZ study was a phase IV, multicentre, open-label study in which patients with moderate-to-severe COPD received indacaterol 150 μg once-daily for 5 months. The primary endpoint was the correlation between immediate response of forced expiratory volume in 1 s (FEV_1_) post-inhalation of salbutamol (400 μg) at study entry and the change from baseline in trough FEV_1_ after 1 month of indacaterol. Secondary endpoints included dyspnoea measured by the modified Medical Research Council (mMRC) grade and health-related quality of life measured by the clinical COPD questionnaire (CCQ).

**Results:**

Of the 602 patients enrolled from 177 centres in France, 543 patients received at least one indacaterol dose, 512 patients completed 1 month of indacaterol treatment (primary endpoint), and 400 patients completed 5 months of treatment. At study entry, mean FEV_1_ values before and after salbutamol inhalation were 1.54 ± 0.50 L and 1.65 ± 0.53 L, respectively. Based on the magnitude of an immediate response of FEV_1_ after salbutamol inhalation at study entry, patients were classified into reversible (Rv, ≥12% and ≥200 mL from pre-salbutamol value; *n* = 106) and non-reversible (NRv, <12% or <200 mL from pre-salbutamol value; *n* = 431) groups. After 1 month of indacaterol treatment, mean absolute and relative difference in trough FEV_1_ were 100 mL and 9%, respectively. No significant correlation was found between the immediate FEV_1_ response to salbutamol at study entry and change from baseline in trough FEV_1_ after 1 month of indacaterol treatment (correlation coefficient = 0.056 [95% CI;−0.032, 0.144] for absolute response and 0.028 [95% CI;−0.06, 0.116] for relative response). At all subsequent visits, mMRC and CCQ scores, and FEV_1_ improved from baseline with no significant difference between the Rv and NRv groups.

**Conclusions:**

Immediate FEV_1_ response to salbutamol did not predict the long-term benefits observed with indacaterol treatment in patients with COPD. Patients considered reversible or non-reversible to salbutamol showed comparable improvements in lung function, dyspnoea and health-related quality of life.

**Trial registration:**

ClinicalTrials.gov: NCT01272362. Date: January 5, 2011

## Background

Chronic obstructive pulmonary disease (COPD) is characterised by persistent airflow limitation that is usually progressive and can lead to dyspnoea and impaired quality of life [1, 2]. The Global Initiative for Chronic Obstructive Lung Diseases (GOLD) strategy recommends long-acting bronchodilators for maintenance treatment in patients with moderate-to-severe COPD, as they have been shown to improve lung function, dyspnoea, quality of life and exacerbation rate [2]. Among inhaled long-acting β_2_-agonists (LABAs), salmeterol and formoterol are administered twice-daily and provide bronchodilation for approximately 12 h [3–6], while indacaterol (Onbrez® Breezhaler®), an ultra-LABA, is administered once daily and provides 24 h of sustained bronchodilation [1].

Bronchodilator reversibility testing, despite not being mentioned in the current treatment recommendations [2], is commonly used in daily clinical practice to predict the usefulness of a bronchodilator treatment [2]. Although the test is simple, it is difficult to interpret or rely on because the response may vary depending on the day and time of testing, severity of baseline lung-function impairment, and the number of drugs given to perform the test [2]. Published data further suggests that acute reversibility in response to bronchodilators in patients with COPD does not predict clinical outcomes [7, 8].

The present study evaluated the correlation between the immediate forced expiratory volume in 1 s (FEV_1_) response with a short-acting β_2_-agonist, salbutamol 400 μg (via a metered-dose inhaler), and the clinical and lung function responses to treatment with indacaterol 150 μg once daily (via the Breezhaler® device) after 1–5 months in patients with moderate-to-severe COPD.

## Methods

### Study design and patients

**Fig. 1:**
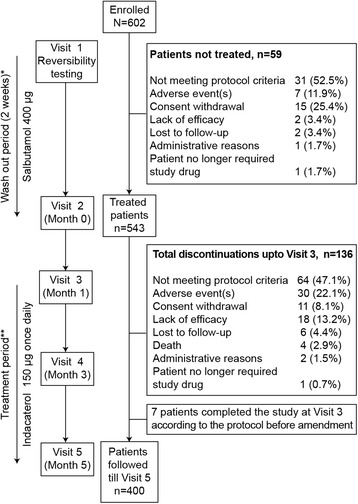
Study flow chart. Data presented are n (%); *During the washout period, patients received salbutamol as a rescue medication; ** All patients received indacaterol

This was a phase IV, multicentre, open-label study conducted at 177 centres in France between December 2010 and May 2012. Men and women aged ≥40 years, with a smoking history of ≥10 pack-years and with moderate to severe COPD [9] as defined by a post-bronchodilator FEV_1_ ≥ 30% and <80% of the predicted value and a post-bronchodilator FEV_1_ to forced vital capacity (FVC) ratio <0.70, were recruited in the study. Patients were excluded if they had a COPD exacerbation within 6 weeks before entering the study, long-term oxygen therapy, asthma, or any concomitant pulmonary disease. Enrolled patients were evaluated at five study visits (Fig. 1). At Visit 1 (study entry), any long-acting bronchodilator treatments were discontinued. Patients who were using inhaled corticosteroids at study entry were allowed to continue using it throughout the study without modifying the drug dose or regimen. The salbutamol reversibility test was performed during which FEV_1_ was measured 30 min after inhalation of four puffs of salbutamol (100 μg each). Patients were then classified into the reversible (Rv) group, defined by a post-bronchodilator increase in FEV_1_ ≥ 12% and ≥200 mL from pre-salbutamol value, or non-reversible (NRv) group, as defined by a post-bronchodilator change in FEV_1_ < 12% or <200 mL from pre-salbutamol value [9–11]. Salbutamol was the only bronchodilator allowed for use during the 2-week washout period. From Visit 2 (baseline) to Visit 5 (at 5 months), indacaterol 150 μg was administered once daily, preferably in the morning and spirometry was performed before inhalation of indacaterol (for measuring trough FEV_1_) at each visit. There was one protocol amendment. During the trial, the committee recommended that 1 month of treatment (till Visit 3) was too short for optimum evaluation of health-related quality of life. The protocol was therefore amended to extend the study duration by 4 months consisting of Visit 4 and Visit 5 (Fig. 1).

### Outcomes

Variables were evaluated at each treatment visit (Visit 2 to Visit 5) and the difference from baseline (Visit 2) was calculated in both the Rv and NRv groups. The primary endpoint of the study was the correlation between the immediate reversibility of FEV_1_ 30 min post-inhalation of salbutamol 400 μg at Visit 1 and the change in trough FEV_1_ after 1 month of indacaterol treatment (between Visit 3 and Visit 2). Absolute FEV_1_ reversibility (expressed in litres) was defined as the difference between FEV_1_ before and after salbutamol administration. Relative FEV_1_ reversibility (expressed as a percentage value) was calculated as (absolute reversibility/FEV_1_ before salbutamol administration) × 100. Secondary endpoints included FEV_1_ change across the study (minimal clinically important difference [MCID] ≥ 100 mL improvement), dyspnoea measured by the modified Medical Research Council (mMRC) grade (MCID decrease of ≥1 point) [12], and health-related quality of life (HRQoL) measured by the clinical COPD questionnaire (CCQ; MCID decrease of ≥0.4 point) [13]. Safety assessments included recording and monitoring of all adverse events (AEs) and serious adverse events (SAEs).

### Statistical analysis

A total of 540 patients were necessary to provide at least 80% power to detect a coefficient of correlation between the immediate reversibility at 30 min post-inhalation in FEV_1_ with salbutamol 400 μg at Visit 1 and the variation in trough FEV_1_ between Visit 3 and Visit 2 of 0.12. Estimating that about 10% of the data would be incomplete, 600 patients were to be included in the study. The intent-to-treat (ITT) population used for evaluation of primary and secondary endpoints included all enrolled patients in the study who received at least one dose of indacaterol 150 μg and for whom at least one endpoint assessment was available after treatment administration. The safety population included all enrolled patients, who received at least one dose of indacaterol 150 μg and for whom at least one tolerability assessment was available after treatment administration. Descriptive statistics were used for all analyses. The 95% confidence intervals (CI) were provided for main assessment criteria. Statistical tests between the Rv and NRv groups (Chi-square or Wilcoxon rank sum test, according to the variables) were bilateral with a significant threshold of 0.05. However, as there was no comparison group in this study, analyses were exploratory and descriptive and *p*-values were provided only as an indication. All analyses were carried out using SAS v8.2.

## Results

Of the 602 patients enrolled, 543 patients received at least one dose of indacaterol, 512 patients completed the study for the primary efficacy endpoint (Visit 3) and 400 patients were followed-up for 5 months (Fig. 1). Of the 195 (32.4%) patients who discontinued the study, 59 patients discontinued between Visit 1 and Visit 2 and did not receive indacaterol; and 136 patients discontinued between Visit 2 and Visit 3. Additionally, 7 patients completed the study at Visit 3 according to the protocol before amendment. Reasons for study discontinuation are listed in Fig. 1.

Clinical characteristics of the treated patients (*n* = 543) are presented in Table 1. The mean age was 63 years, and most patients were men (74%) and ex-smokers (64%). The mean ± SD FEV_1_ values before and after salbutamol inhalation were 1.54 ± 0.50 L and 1.65 ± 0.53 L, respectively. Of the 543 treated patients, 6 patients had non-interpretable reversibility results. In patients with interpretable reversibility results, 106 patients (20%, Rv group) showed significant FEV_1_ reversibility with salbutamol, and 431 patients (80%, NRv group) showed non-significant reversibility. Baseline demographics and patient characteristics were comparable between the Rv and NRv groups (Table 1) and there was no difference in the use of inhaled corticosteroids between these groups.

**Table 1:** Demographics and baseline characteristics of patients who received at least one dose of indacaterol

Variables	Total population	Rv group	NRv group
	(*N* = 543)^a^	(*n* = 106)	(*n* = 431)
Age, years	62.7 (9.6)	60.8 (9.9)	63.2 (9.5)
Male, *n* (%)	404 (74.4)	80 (75.5)	319 (74.0)
Current smoker, *n* (%)	196 (36.1)	43 (40.6)	151 (35.0)
Number of pack years	40.3 (19.3)	42.2 (20.7)	39.8 (19.0)
Years with COPD	9 (7.4)	9.9 (8.2)	8.8 (7.1)
Post-bronchodilator FEV_1_ (L)	1.65 (0.53)	1.88 (0.51)	1.59 (0.52)
Post-bronchodilator FEV_1_ (L), % predicted	57.3 (14.12)	62.8 (12.24)	55.9 (14.23)
Post-bronchodilator FVC (L)	3.01 (0.82)	3.36 (0.84)	2.92 (0.80)
FEV_1/_FVC (%) (Visit 2)	53.5 (14.15)	53.3 (10.80)	53.4 (14.89)
mMRC score (Visit 2)	1.4 (0.84)	1.3 (0.85)	1.4 (0.84)
CCQ score (Visit 2)	2.0 (0.99)	1.9 (0.93)	2.0 (1.01)

^a^ 6 patients had non interpretable reversibility data; Data are presented as mean (SD) unless otherwise specified; CCQ, Clinical COPD Questionnaire; COPD, chronic obstructive pulmonary disease; FEV_1_, forced expiratory volume in 1 second; FVC, forced vital capacity; mMRC, medical Modified Research Council; SD, standard deviation

**Fig. 2:**
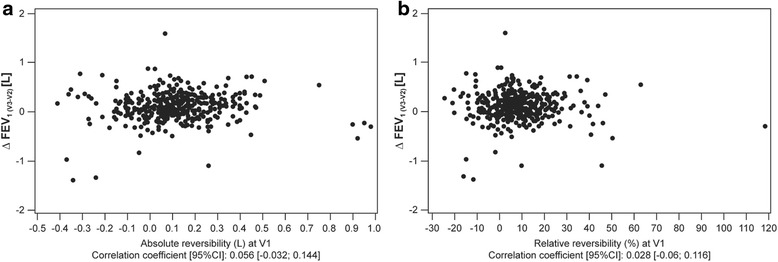
Correlation between ∆ trough FEV_1_ at 1 month (Visit 3-Visit 2) with indacaterol therapy and **a** absolute FEV_1_ reversibility and **b** relative FEV_1_ reversibility to salbutamol at Visit 1. CI, confidence interval; FEV_1,_ forced expiratory volume in 1 s; V1, Visit 1; V2, Visit 2; V3, Visit 3

**Fig. 3:**
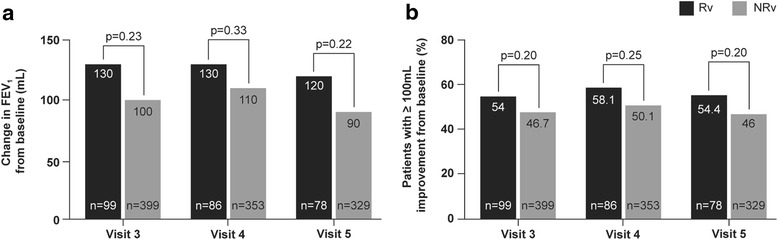
**a** Mean change in trough FEV_1_ (ml) from baseline and **b** proportion of patients reaching MCID for FEV_1_ during treatment with indacaterol in reversible (Rv) and non-reversible (NRv) groups. FEV_1,_ forced expiratory volume in 1 s; MCID, minimal clinically important difference; NRv, non-reversible groups; Rv, reversible groups

In patients who received indacaterol, mean absolute and relative improvements in FEV_1_ after 1 month of indacaterol treatment (Visit 3-Visit 2) were 100 (±260) mL and 9 (±19) %, respectively. No statistically significant correlation was observed between the change in trough FEV_1_ at 1 month and absolute or relative reversibility with salbutamol at study entry (Fig. 2a and b). Improvements in trough FEV_1_ from baseline was observed in both the Rv and NRv groups at all visits (Fig. 3a) with no statistically significant difference between the groups at any visit. The proportion of patients reaching MCID (≥100 mL improvement) for FEV_1_ at each visit was comparable in both groups (Fig. 3b).

**Fig. 4:**
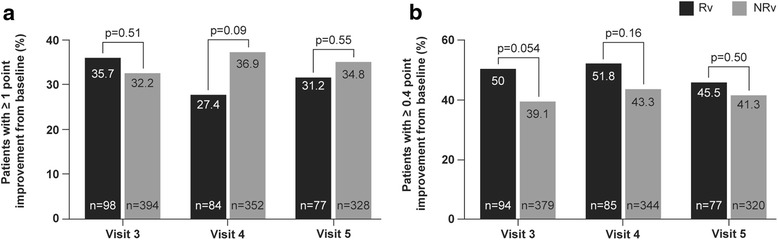
Proportion of patients achieving (**a**) ≥1 point improvement in mMRC score and **b** ≥0.4 point improvement in CCQ scores during treatment with indacaterol. CCQ, clinical COPD questionnaire; mMRC, modified Medical Research Council; NRv, non-reversible groups; Rv, reversible groups

Dyspnoea as measured by mMRC (mean difference in mMRC ranged from−0.3 to−0.2, *data not shown*) improved in both the Rv and NRv groups at all visits, and there were no statistically significant differences between these groups at any visit. The percentage of patients with a ΔmMRC ≥1 from baseline was comparable between the Rv and NRv groups at all visits (Fig. 4a). CCQ scores improved from baseline in both the Rv and NRv groups at all visits with no statistically significant differences between the groups at any visit (*data not shown*). The percentage of patients reaching the MCID in terms of ΔCCQ ≥ 0.4 (Fig. 4b) from baseline was not significantly different between the two groups at any visit.

Of the 543 patients included in the safety analysis, 264 (48.6%) and 33 (6.1%) patients experienced at least one AE and SAE, respectively. Fifty-two (10%) patients reported at least one AEs that was suspected to be related to the study medication: Rv, *n* = 15 (14.2%) vs. NRv, *n* = 37 (8.6%), *p* = 0.10. Among the AEs (i.e., with a frequency > 2%) COPD exacerbations, bronchitis, and rhinitis were not different between the Rv and NRv subgroups (*p* > 0.40). Compared to the NRv group, patients in the Rv group experienced cough more frequently (9.4% vs 4.6%; *p* = 0.02) and a trend towards more frequent dyspnoea (8.5% vs 4.6%; *p* = 0.07). The incidence of SAEs were low and comparable between the Rv and NRv groups: Rv, *n* = 6 (5.7%) vs NRv, *n* = 27 (6.3%). Overall, four deaths were reported in the study. Two of these deaths (one death after a COPD exacerbation and one sudden death) were assessed by an investigator as suspected to be related to study treatment. The other two deaths (one sudden death because of an unknown cause, and one suicide) were determined as not related to study treatment.

## Discussion

The present study showed no correlation between the immediate FEV_1_ response with the short-acting β_2_-agonist salbutamol and the change in trough FEV_1_ after 1-month of treatment with indacaterol 150 μg in patients with moderate-to-severe COPD. When patients were subdivided into the Rv and NRv groups (based on response to salbutamol at study entry), there was no significant difference in the improvement in trough FEV_1_ after 1 month of treatment with indacaterol. Similarly, no statistically significant difference was observed between the Rv and NRv groups over the 5 months of this study. Although numerical improvements in mMRC and CCQ scores from baseline occurred in both groups, no clinically significant difference was observed between the Rv and NRv groups at any visit. These results suggest that salbutamol responsiveness does not predict the long-term lung function or clinical responses to indacaterol.

These findings are consistent with a previous study with another LABA (formoterol) that also reported no significant correlation between reversibility with salbutamol and clinical efficacy after 1 month of treatment in patients with stable COPD [7]. Similar results were reported after treatment with a long-acting antimuscarinic agent in the Understanding Potential Long-term Improvements in Function with Tiotropium (UPLIFT) trial, which demonstrated that acute bronchodilator responsiveness at baseline did not predict long-term clinical response to tiotropium after 4 years of treatment [14]. Tashkin et al. showed that irrespective of the presence of short term response to tiotropium on day 1, treatment with tiotropium over a 1-year period resulted in significant improvement in FEV_1_ compared to baseline in both the responsive and poorly responsive groups, with significant but low correlation (r = 0.43) at the end of the study [15]. Our data corroborates the previous findings by showing the absence of a relationship between acute effects of salbutamol and long term effects of indacaterol, confirming that acute bronchodilator responsiveness should not be used to predict the effects of long-acting bronchodilators.

Results from the present study concur with the GOLD strategy and the other discussed studies [7, 14, 15], acknowledging that the magnitude of reversibility of airflow limitation following inhaled short-acting bronchodilators does not predict the response to long-term treatment with bronchodilators [9]. Non-reversibility of airflow limitation has been used as an inclusion criterion in many COPD studies until recently, however analyses of results from several large studies have concluded that reversibility in COPD patients is a continuous variable. Further, results of reversibility testing vary between tests, time of test, and baseline values and thus should not be used to select patients in a clinical trial or for determining the treatment [16]. It has been also been shown that bronchodilator reversibility is not reproducible over time and does not predict disease progression or mortality [7]. In addition, the current thresholds for spirometric measurements for reversibility are also debatable. Some authors argue that these tests may produce false positive results when the baseline FEV_1_ value is low, and that FEV_1_ measurements are not always repeatable due to noise effects that can be >100 mL [13, 14, 16–18]. However, others have suggested a lower threshold than the fixed 12% and 200 mL European Respiratory Society (ERS)/American Thoracic Society (ATS) criteria, particularly in patients with low baseline FEV_1_ [10]. Pellegrino et al. suggested that any threshold for reversibility testing will have pros and cons, since most proposed thresholds are within the natural variability of FEV_1_ and may not be applicable to all disease conditions [19]. Although, the present findings are not in favour of recommending spirometry use for predicting clinical response, there is an ongoing debate around the use of spirometry to assess bronchodilator response [9, 19, 20]. Till date, only short-acting bronchodilators (e.g. salbutamol) are used for immediate reversibility testing, and we cannot exclude a significant relationship between FEV_1_ change at first dose of indacaterol, due to its rapid onset of action, and long term response, as demonstrated with tiotropium [15].

In this study, indacaterol was well tolerated with only 6.1% of patients reporting at least one SAE. The safety profile was consistent with previous indacaterol studies with approximately 5% of patients experiencing cough. Interestingly, cough was more prevalent in patients in the Rv group compared with the non-Rv group. Although these findings require confirmation, they suggest that bronchial responsiveness may favour cough induced by indacaterol. Of the four deaths reported in the study, two (one death of unknown cause and one following COPD exacerbation) were suspected to be related to the study drug. Sudden deaths are not rare in patients with COPD, and severe COPD exacerbations may be fatal [21, 22].

The strengths of the present study are that the response to indacaterol was studied by measuring FEV_1_ and validated tools (mMRC scale and CCQ questionnaire) for measuring patient-related outcomes under real-life conditions. We also recognise some limitations to this approach. Although the mMRC scale is easy to use and is widely recommended for evaluating dyspnoea in COPD patients in daily practice [9], it has the major disadvantage of being poorly responsive to therapeutic interventions, including bronchodilators and rehabilitation. This was also the case in the present study, despite a significant proportion of responders, when assessed by using the CCQ and MCID. CCQ appears more responsive than the mMRC scale in this real-life study. A comprehensive symptom assessment is also recommended in addition to measuring breathlessness, using either the COPD assessment test (CAT) or CCQ [9], but the knowledge regarding responsiveness of these short questionnaires to pharmacotherapy remains limited. Interestingly, the percentage of responders for HRQoL in the present real-life study was close to those in the active treatment arms of placebo-controlled studies using the reference St. George Respiratory Questionnaire (SGRQ) [23] [24], confirming the validity of these findings in this non-placebo-controlled trial. The goal of the present study was to evaluate the effects of indacaterol in daily practice in symptomatic patients with post-bronchodilator FEV1/FVC < 0.70, the diagnostic criteria for COPD according to the GOLD document. Because FEV1/FVC ratio usually declines with age, some subjects with FEV1/FVC <0.7 have a FEV1/FVC above the lower limit of the age-related predicted value (also called lower limit of normal, LLN) and may not be affected by COPD [25]. Specifically-designed studies with sufficient statistical power will be necessary to examine the effect of bronchodilators on symptoms in these latter patients. Finally, there were high rates of study discontinuation, mostly due to violation of inclusion criteria and withdrawal of patient consent. Such findings are usual in phase IV studies and are unlikely to affect our conclusions.

## Conclusion

In patients with COPD treated with indacaterol for 5 months, immediate FEV_1_ reversibility with salbutamol at baseline did not predict the sustained response to indacaterol in terms of lung function, health-related quality of life or dyspnoea.

## Abbreviations

AE: Adverse event
CAT: COPD assessment test
CCQ: Clinical COPD questionnaire
CI: Confidence interval
COPD: Chronic obstructive pulmonary disease
FEV_1_: Forced expiratory volume in 1 s
FVC: Forced vital capacity
GOLD: Global initiative for chronic obstructive lung diseases
HRQoL: Health-related quality of life
ITT: Intent-to-treat
LABAs: Long-acting β_2_-agonists
MCID: Minimal clinically important difference
mMRC: Modified medical research council
NRv: Non-reversible
Rv: Reversible
SAE: Serious adverse event
SGRQ: Saint George respiratory questionnaire
UPLIFT: Understanding potential long-term improvements in function with Tiotropium
